# Epidemiology of *Taenia saginata* taeniosis/cysticercosis: a systematic review of the distribution in the Middle East and North Africa

**DOI:** 10.1186/s13071-019-3339-5

**Published:** 2019-03-15

**Authors:** Anastasios Saratsis, Smaragda Sotiraki, Uffe C. Braae, Brecht Devleesschauwer, Veronique Dermauw, Ramon M. Eichenberger, Lian F. Thomas, Branko Bobić, Pierre Dorny, Sarah Gabriël, Lucy J. Robertson

**Affiliations:** 1 Veterinary Research Institute, Hellenic Agricultural Organization Demeter, Thermi, 57001 Greece; 2 One Health Center for Zoonoses and Tropical Veterinary Medicine, Ross University School of Veterinary Medicine, Basseterre, Saint Kitts and Nevis; 3 Department for Infectious Disease Epidemiology and Prevention, Statens Serum Institut, Copenhagen, Denmark; 4 Department of Epidemiology and Public Health, Sciensano, Ixelles, Brussels Belgium; 5 Department of Veterinary Public Health and Food Safety, Ghent University, Merelbeke, Belgium; 6 Department of Biomedical Sciences, Institute of Tropical Medicine, Antwerp, Belgium; 7 Institute of Parasitology, Vetsuisse Faculty, University of Zurich, Zurich, Switzerland; 8 Institute of Infection & Global Health, University of Liverpool, IC2 Building, 146 Brownlow Hill, Liverpool, L3 5RF UK; 9 International Livestock Research Institute, PO Box 30709, Nairobi, 00100 Kenya; 10 Centre of Excellence for Food- and Vector-borne Zoonoses, Institute for Medical Research, University of Belgrade, Belgrade, Serbia; 11 Department of Virology, Parasitology and Immunology, Ghent University, Merelbeke, Belgium; 12 Department of Food Safety and Infection Biology, Faculty of Veterinary Medicine, Norwegian University of Life Sciences, Sentrum, PO Box 369, 0102 Oslo, Norway

**Keywords:** Beef tapeworm, Bovine cysticercosis, Cestode, Foodborne parasites, Middle East, MENA, North Africa, *Taenia saginata*, Taeniosis

## Abstract

**Background:**

The zoonotic parasite *Taenia saginata* utilizes bovines as an intermediate host (causing cysticercosis) and humans as the definitive host (causing taeniosis). The public health burden of *T. saginata* is assumed to be low, but the economic burden is large, due to the resources utilized in the detection and condemnation of infected carcasses and carcass parts. As part of a collaborative effort to synthesize worldwide epidemiological data on this parasite, we present here the results of a systematic review on the distribution of *T. saginata* taeniosis and bovine cysticercosis in the Middle East and North Africa (MENA).

**Methods:**

Information on the occurrence and prevalence of *T. saginata* taeniosis and cysticercosis in the MENA region was obtained through a systematic review of published and grey literature, including OIE reports, published between January 1st, 1990 and December 31st, 2017.

**Results:**

A total of 63 publications were retrieved across the 21 MENA countries. *Taenia saginata* taeniosis was reported in 11 of these countries, whereas unspecified taeniosis was reported for a further seven. Microscopy-based prevalence values ranged between 0.02–8.6%. Bovine cysticercosis prevalence estimates based on meat inspection were only reported for Egypt and Israel, with prevalence data ranging between 0.2–20% and 0.1–9.1% for cattle and buffaloes, respectively. The presence of bovine cysticercosis could be confirmed for 10 additional countries through OIE reports.

**Conclusions:**

Human taeniosis occurrence was confirmed for 86% (18/21) of the countries in the MENA region, although in several of these countries the species responsible was not specified. Religious prohibitions on the consumption of pork and the limited extent of pig farming across much of this region, however, suggest that many reported taeniosis cases are likely to be attributable to *T. saginata* rather than *Taenia solium* or *Taenia asiatica*. There was a paucity of data regarding both the prevalence and economic impact of bovine cysticercosis. More detailed epidemiological data on both *T. saginata* taeniosis and bovine cysticercosis could be obtained by adopting an integrated “One Health” approach, considering the characteristics (e.g. ecosystem related and sociopolitical aspects) of the MENA region. Compared with more conventional approaches, this could lead to an enhanced performance and cost-effectiveness of surveillance systems.

**Electronic supplementary material:**

The online version of this article (10.1186/s13071-019-3339-5) contains supplementary material, which is available to authorized users.

## Background

The Middle East and North Africa (MENA) is situated at the natural crossroads of three continents and has significantly contributed to the development of flourishing civilizations, the expansion of maritime empires, and the spread of three of the major religions of the world [1]. Early animal domestication in the area, which, based on Neolithic fossils, dates back to 6000 B.C., led to an early close relationship between people and domestic animals, providing an ideal interface for the development of zoonoses [2]. Indeed, paleoparasitological studies in the area have confirmed that zoonoses (e.g. taeniosis) were established here in ancient times [3–5]. In addition, in recent decades numerous zoonoses have emerged or re-emerged in this part of the world [6–9], which to some extent can be explained by socioeconomic changes, conflicts, and political instability, all of which have resulted in fragile healthcare systems (limited laboratory and clinical capacities), increased human and animal mobility (travel, displacement, and lack of stringent animal import regulations), gaps in the knowledge of risk factors for transmission of emerging infections, and surveillance systems being unable to address early detection and rapid response. Furthermore, climate change-driven ecosystem fragility (arid regions, desertification, water scarcity) further impacts the situation [10]. Additionally, some of the petroleum-rich countries in the Arabian Peninsula represent attractive migratory destinations for tens of millions of economic migrants from neighboring regions such as South Asia or East Africa [11].

*Taenia saginata*, the beef tapeworm, is an important cyclo-zoonotic cestode with a worldwide distribution. The hermaphrodite adult tapeworm develops in the human intestine and produces tens of thousands of eggs that are either excreted free or within intact, motile, proglottids in the faeces [12]. The eggs are able to survive for several months in the environment [13]. Bovids, typically cattle and buffaloes, which are of particular importance in the MENA region, are the natural intermediate hosts of the parasite, and are infected by ingestion of eggs. The oncosphere migrates *via* the bloodstream to striated muscles, where the metacestode larval stage (cysticercus) develops. The success and widespread distribution of this parasite can be associated with a range of factors including dietary habits (consumption of raw or undercooked cysticerci-infected meat), leisure activities in close proximity to grazing areas, free access of cattle to surface water, and sanitary education level of the farm workers, as well as inadequate treatment and disposal of sewage [14–17].

Although *T. saginata* taeniosis is characterized by mild, or absence of clinical symptoms, rare complications such as gangrenous cholecystitis, cholangitis, abdominal discomfort and acute appendicitis have been described (reviewed in [18]). Thus, the major burden of this parasite is upon the meat industry, where considerable economic losses occur due to the cost of meat inspection, carcass condemnation in cases of heavy infections, and obligatory freezing, heating, or irradiation in cases of light infections, along with additional transport or even insurance costs in some countries [19, 20]. The bovine population of the MENA region is huge, with Sudan, Egypt, Algeria, Yemen, and Syria, sorted by population rank in descending order based on 2016 data, counting among the top-producing countries in the region with an approximate population reaching almost 45 million head (including buffaloes, which are of relevance for Egypt), more than 66% of which are kept in Sudan [21]. Both traditional and modernized bovine production systems are found in the MENA region. The traditional systems mainly cater for nomadic producers (extensive production system/mainly meat oriented) or producers who have settled in close vicinity to cities/irrigated agricultural areas and rely on crop residues. Modernized systems largely serve intensively reared dairy cattle of both local and imported breeds [22].

This review provides a systematic overview of the epidemiology of *T. saginata* and bovine cysticercosis in the MENA region. To the best of our knowledge, an article addressing this cestode in the MENA region has not previously been published.

## Methods

### Search strategy

This systematic review was conducted according to the PRISMA guidelines (Additional file 1) and focused on the region of Middle East and North Africa [23], specifically the countries: Algeria, Cyprus, Egypt, Iraq, Israel, Jordan, Kuwait, Lebanon, Libya, Morocco, Oman, Palestine, Qatar, Saudi Arabia, South Sudan, Sudan, Syria, Tunisia, United Arab Emirates (UAE), Western Sahara and Yemen. It utilized records relating to the occurrence, prevalence, and geographical distribution of human taeniosis due to *T. saginata* and bovine cysticercosis for the period between the 1st of January 1990 and 31st of December 2017. A specific combination of search words was used to search both for published papers and grey literature (MSc/PhD theses, reports etc.) in two international bibliographic databases (PubMed and opengrey.eu). The search term was as follows: (cysticerc* OR cisticerc* OR “C. bovis” OR taenia* OR tenia* OR saginata OR taeniosis OR teniosis OR taeniasis OR ténia OR taeniid OR cysticerque) AND (above-mentioned countries separated by the operator “OR”). In addition, WHO IRIS (http://apps.who.int/iris/) and Index Medicus for the Eastern Mediterranean Region (IMEMR) (http://www.emro.who.int/information-resources/imemr-database/) were searched by using a combination of three search words (i.e. *Taenia* and *saginata* or cysticercus), which cannot be further extended due to the limitation in the number of search words to be used by those databases. A late stage search was also conducted using the Google search engine and aimed specifically at trying to identify documents for countries for which the previous approaches had provided no or only very few records. Finally, data on both occurrence and number of bovine cysticercosis cases, whenever available, were also retrieved from OIE interfaces HANDISTATUS II (http://web.oie.int/hs2/report.asp?lang=en), and WAHIS (http://www.oie.int/wahis_2/public/wahid.php/Diseaseinformation/statusdetail), which provide data for the periods between 1994–2004 and 2005 until the end of the study period, respectively.

### Selection criteria, data extraction

Retrieved records were first screened to exclude duplicates. Subsequently, titles and abstracts of all unique records were screened for their relevance to the scope of the review. This was done based on the following list of exclusion criteria (Additional file 2): (i) publication date before 1990 or after 2017; (ii) studies concerning a parasite other than *T. saginata*; (iii) studies reporting data from countries other than those listed in the MENA region; (iv) studies providing information other than the scope of the review question (occurrence, prevalence, and geographical distribution of *T. saginata* taeniosis and bovine cysticercosis).

If it was not possible to determine the eligibility of a document from the abstract and title only, the full text was screened. Full texts, including relevant citations therein, were then retrieved where possible and evaluated by the same criteria as above. Data were extracted into tables that are presented in Tables 1–4. Prevalence data were only extracted if both the numerator and the denominator were provided, and 95% confidence intervals were calculated based on the Clopper and Pearson method.

## Results

### Search results

**Fig. 1:**
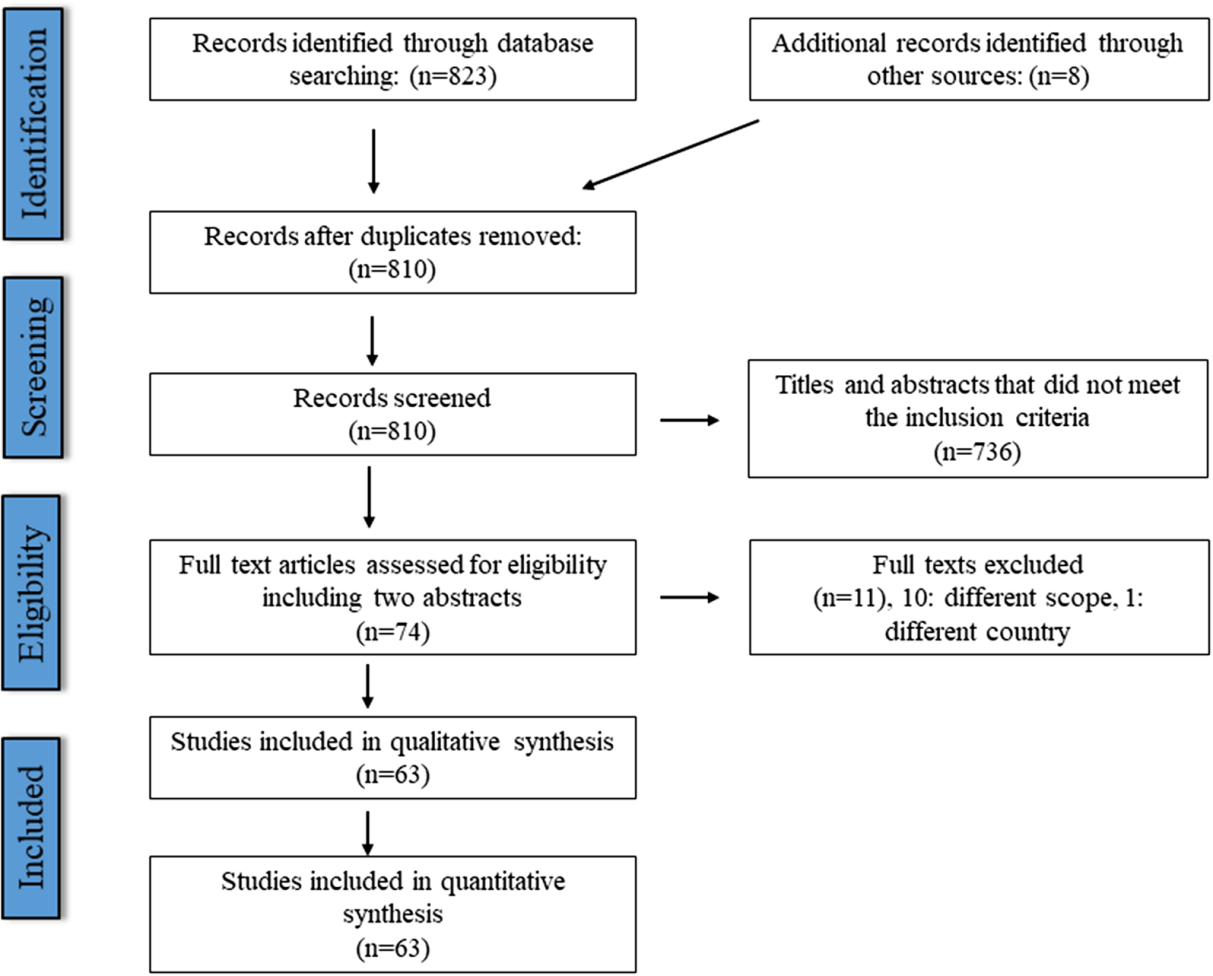
PRISMA flow chart

Literature search of all four databases for the 21 MENA countries returned 823 results, of which 21 were duplicates (Fig. 1). Subsequent screening of titles and abstracts limited the number of records to 55 in line with the selection criteria. For two of these, full texts could not be retrieved. However, data in the abstracts of these articles were sufficient for prevalence calculation. Eight records were additionally retrieved through citations in the above papers (4 records) and the late stage search (4 records), resulting in a total number of 63 records to be screened. A total of 58 records reported on taeniosis presence/prevalence and 8 on bovine cysticercosis prevalence, with 3 of them reporting on both. However, data regarding human taeniosis in one of the above three records were inconsistent and could not be considered. Most studies were from Egypt (*n* = 19), followed by Sudan (*n* = 7), Lebanon (*n* = 6), and Saudi Arabia (*n* = 5).

### Human taeniosis occurrence

Of the 58 records reporting the presence/prevalence of taeniosis, 5 were case reports, whereas 53 reported infection prevalence in particular study groups, such as schoolchildren, immigrants, refugees, housemaids, food handlers, or groups presenting a certain health condition (e.g. abdominal pain, diarrhea, appendicitis) and its relation to parasitism. The age of participants ranged between 1–90 years. Diagnosis was based mainly on microscopy of stool samples (e.g. wet smears, concentration and/or flotation methods) and/or microscopy-based proglottid identification. However, it was not always apparent from the articles if and how species identification was performed. In a single record from Egypt, *T. saginata* was confirmed using molecular methods [24].

**Fig. 2:**
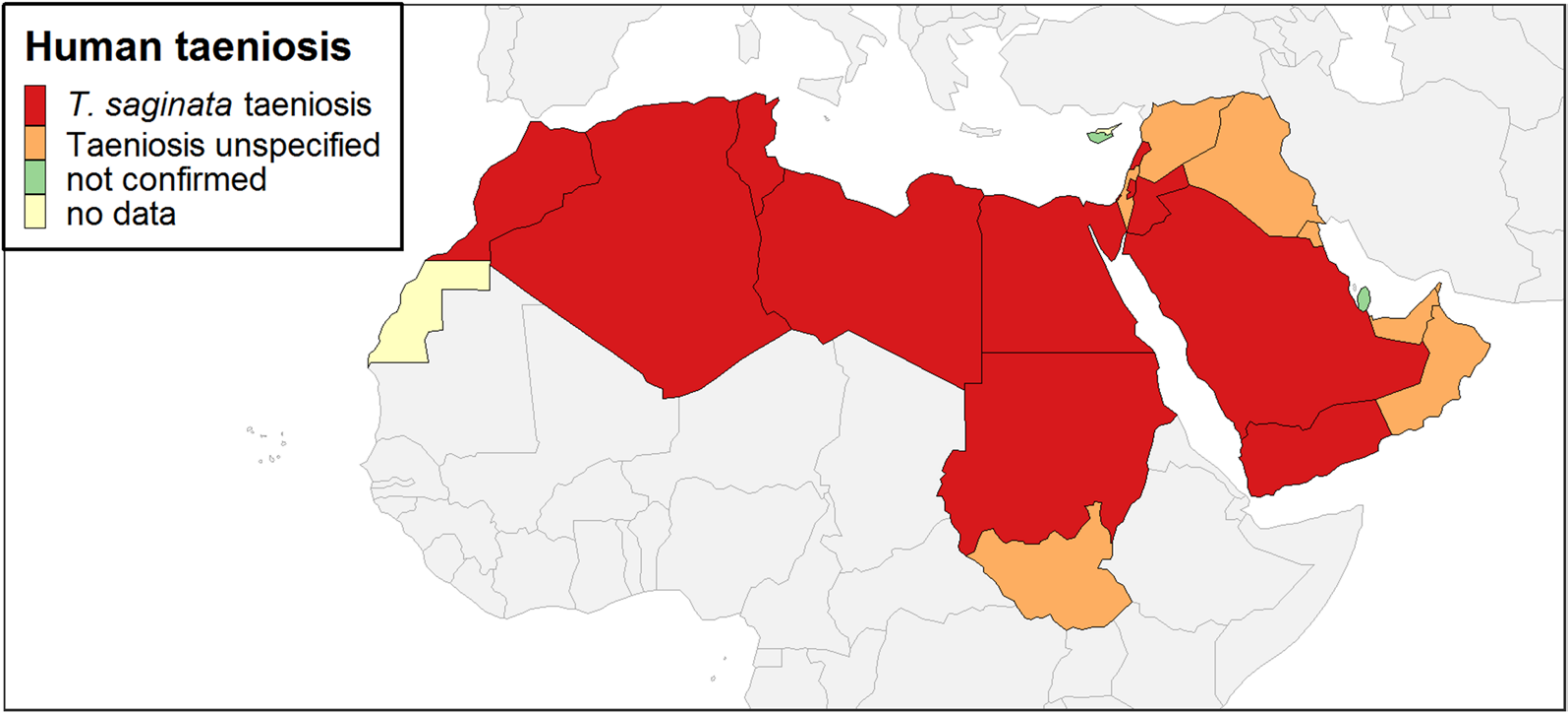
Countries with reports of taeniosis due to *Taenia saginata* and *Taenia* spp. in the period 1990–2017

Individual case reports confirmed the presence of *T. saginata* taeniosis in Lebanon, Morocco and Sudan, and an unspecified taeniosis case was described from Syria (Table 1). The remaining population-based prevalence studies confirmed the occurrence of unspecified taeniosis in seven countries, namely Iraq, Israel, Kuwait, Oman, South Sudan, Syria and UAE (Table 2). *Taenia saginata* taeniosis was reported from the following countries (11 in total): Algeria, Egypt, Jordan, Lebanon, Libya, Morocco, Palestine, Saudi Arabia, Sudan, Tunisia and Yemen (Table 2). *Taenia* spp. infections were not detected in two studies from Qatar with sample sizes of 1737 and 9208 respectively, whereas in the Republic of Cyprus, *T. saginata* is considered to be eliminated [25]. For Western Sahara, relevant data could not be retrieved from the databases. Thus, evidence for the presence of human *Taenia* spp. infections could be found for 18 out of 21 MENA countries for the study period between 1990 and 2017, with 11 of them specifically indicating *T. saginata* infections (Fig. 2). Microscopy-based prevalence values ranged between 0.02–8.6%.

**Table 1:** *Taenia saginata* or *Taenia* spp. taeniosis case reports

Country (city)	No. of cases	Age	Species	Diagnosis	Reference
Lebanon (Tripoli)	1	69	*T. saginata*	Proglottid identification of the part of tapeworm found in the peritoneal exudate after jejunal perforation	[61]
Morocco (Rabat)	1	63	*T. saginata*	Tapeworm detection during capsule endoscopy. Proglottid identification after post-treatment elimination	[62]
Sudan (Khartoum)	1	50	*T. saginata*	Part of a tapeworm found in jejunostomy leak after esophagectomy	[63]
Sudan (flame)	1	43	*T. saginata*	Proglottid identification of vomited part of a tapeworm	[64]
Syria (Allepo)	1	70	*Taenia* spp.	Tapeworm detection during esophagogastroduodenoscopy	[65]

**Table 2:** Prevalence of taeniosis in humans based on cross-sectional or retrospective studies

Country	Timeframe	Location of study	Age range tested	No. positive/total no. of people tested	Prevalence (%) (95% CI)	Species	Technical diagnosis	Ref
Algeria	12/2010–11/2011	Oran	1–80	1/1042	0.1 (<0.01–0.5)	*T. saginata*	Microscopy	[66]
Egypt	on	Sohag (Sohag Governorate)	12–90	5/150	3.2 (1.0–7.3)	*Taenia* spp.	Microscopy	[67]
Egypt	09/2013–08/2014	Sohag (Sohag Governorate)	1–14	1/100	1.0 (0.03–5.5)	*Taenia* spp.	Microscopy	[68]
Egypt	01/2009–12/2009	Qalyubia, Dakahlia and Damietta Governorates	1– >40	2/105	1.9 (0.2–6.7)	*Taenia* spp.	Microscopy	[69]
Egypt	12/2005–12/2006	Mansoura (Dakahlia Governorate)	on	37/3180	1.1 (0.8–1.6)	*T. saginata*	Microscopy	[70]
Egypt	01/2005–01/2006	Mansoura and Gogar (Dakahlia Governorate)	20–40	2/2000	0.1 (<0.01–0.4)	*T. saginata*	Microscopy (including proglottid identification)	[71]
Egypt	on	Qalyub (Qalyubia Governorate)	6–12	2/486	0.4 (0.05–1.5)	*T. saginata*	Microscopy	[72]
Egypt	on	Sennores (El-Fayum Governorate)	6–12	3/252	1.2 (0.3–3.4)	*T. saginata*	Microscopy (including proglottid identification)	[73]
Egypt	on	Ashmoun, Tala, Berket El Sabaa Shebeen El Koom, Menouf (Menoufia Governorate)	<10– >50	2/565	0.4 (0.05–1.4)	*Taenia* spp.	Microscopy	[74]
Egypt	05/2006–06/2007	El-Eman, El-Matieea, El-Ezeia (Assiut Governorate)	on	2/325	0.6 (0.07–2.2)	*T. saginata*	Microscopy	[29]
Egypt	01/2001–12/2008	Alexandria, Ismailia (Alexandria and Ismailia Governorates)	1–17	8/1500	0.5 (0.2–1.0)	*T. saginata*	on	[75]
Egypt	on	El-Ghanayem (Assiut Governorate)	6–11	1/400	0.3 (<0.01–1.4)	*T. saginata*	on	[76]
Egypt	01/2014–12/2014	Benha (Qalyubia Governorate)	20–55	6/100	6.0 (2.2–12.6)	*T. saginata*	Microscopy (including proglottid identification) and molecular confirmation	[24]
Iraq	04/1988–03/1989	Kirkuk area (Al-Tameem/Kirkuk Governorate)	6–12	9/1681	0.5 (0.25–1.0)	*Taenia* spp.	Microscopy	[77]
Israel	on	on	on	3/93	3.2 of ^a^ (0.7-9.1)	*Taenia* spp.	on	[78]
Israel	2007–2011	Beer Sheva (Negev region)	0–19	8/45,978	0.02^b^ (0.008-0.03)	*Taenia* spp.	Microscopy	[79]
Jordan	2009–2013	Irbid, Jerash and Ajlun	All age groups	48/21,906	0.2 (0.2–0.3)	*Taenia* spp.	Microscopy	[80]
Jordan	07/1987–07/1988	Irbid	on	1/283	0.4 (<0.01–2.0)	*T. saginata*	Microscopy	[81]
Kuwait	on	Kuwait	1– >40	1/1674	0.06 (<0.01–0.3)	*Taenia* spp.^c^	Histology of appedenctomy sections	[82]
Kuwait	01/1986–12/1986	Six general hospitals (Adan, Amiri, Mubarak, Jahra, Farwaniya and Infectious Diseases)	1–69	17/6000	0.3 (0.2–0.5)	*Taenia* spp.	Microscopy	[83]
Lebanon	01/1997–12/1998 & 01/2007–12/2008	Beirut	on	116/14,771	0.8^d^ (0.7–0.9)	*Taenia* spp.	Microscopy	[84]
				27/7477	0.4^e^ (0.2-0.5)			
Lebanon	1997–2001	Tripoli	<5– >66	188/17,126	1.1 (1.0–1.3)	*Taenia* spp.	Microscopy	[85]
Lebanon	on	Tripoli, Beirut	on	2479/44,864	5.5 (5.3–5.7)	*Taenia* spp.	Microscopy	[86]
Lebanon	1995–1997	Beirut	14–71	on	Either 0.8 (0.5–1.3) or 0.4^f^	*Taenia* spp	Microscopy	[87]
Lebanon	05/2004–09/2004	North Lebanon (Akkar Governorate)	16–50	1/308	0.3 (<0.01–1.8)	*Taenia* spp.	Microscopy	[88]
Libya	03/2004–06/2004	Tripoli	5–18	1/50	2.0 (0.05–10.7)	*T. saginata*	Microscopy	[89]
Morocco	1996–2005	Kenitra	on	6/4285	0.14 (0.05–0.3)	*T. saginata*	Microscopy	[90]
Morocco	on	Me Mellal	7–14	4/740	0.5^g^ (0.2–1.4)	*Taenia* spp	Microscopy	[48]
				0/603	0^h^ (0-0.6)			
Oman	09/2004–03/2005	Dhahira Governorate	9–10	8/436	1.8^i^ (0.8-3.6)	*Taenia* spp	Microscopy	[91]
Oman	on	Dhofar Governorate (Dhalqut, Rakhyut, Salalah, Taqah, Mirbat Wilayats)	All age groups	0/5253	0 (0–0.07)	*Taenia* spp.	on	[92]
Palestine	01/1998–12/2007	Gaza strip (Gaza, North, Mid-Zone, Khan Younis and Rafah Governorates)	All age groups	on	<1.0^j^	*T. saginata*	Microscopy	[49]
Palestine	11/2002–04/2003	Khan Younis Governorate	6–11	0/1370	0 (0–0.3)	*Taenia* spp.	Microscopy	[93]
Qatar	01/2005–12/2006	Doha	15–50	0/1737	0^k^	*Taenia* spp.	Microscopy	[94]
Qatar	01/2005–12/2008	Doha	1–80	0/9208	0	*Taenia* spp.	Microscopy	[95]
Saudi Arabia	09/2012–12/2012	Hail	on	2/130	1.5 (0.2–5.5)	*Taenia* spp.	Microscopy	[96]
Saudi Arabia	01/2010–12/2010	Hofuf, Khobar, Damman and suburban areas	2–18	5/1600	0.3 (0.1–0.7)	*T. saginata*	on	[97]
Saudi Arabia	10/2009–01/2011	Al-Baha	on	119/2000	6.0^l^ (5.0-7.0)	*T. saginata*	Microscopy	[98]
Saudi Arabia	2012	Madinah	20–55	1/120	0.8^m^ (0.02-4.6)	*Taenia* spp.	Microscopy	[99]
Saudi Arabia	01/1990–12/1992	Abha	17–45	0/5518	0^n^ (0–0.07)	*Taenia* spp.	Microscopy	[100]
South Sudan	on	Already	4– >50	1/241	0.4 (0.01–2.3)	*Taenia* spp.	Microscopy	[101]
Sudan	11/2003–10/2005	Khartoum	<21– >51	4/1500	0.3^o^ (0.07-0.7)	*T. saginata*	Microscopy	[102]
Sudan	03/1990–02/1991	Khartoum	<5	5/298	1.7 (0.6–3.9)	*T. saginata*	Microscopy	[103]
Sudan	12/2016–04/2017	Khartoum	5–14	0/120	0 (0–3.0)	*Taenia* spp.	Microscopy	[104]
Sudan	01/2013–06/2013	Khartoum	1–5	6/562	1.1 (0.4–2.3)	*T. saginata*	Microscopy (including proglottid or scolex identification)	[105]
Sudan	on	Khartoum	Primary school children	43/500	8.6 (6.3–11.4)	*T. saginata*	Microscopy	[106]
Syria	03/2006–06/2006	Damascus	6–12	0/1469	0 (0–0.3)	*Taenia* spp.	Microscopy	[107]
Tunisia	01/1997–12/2006	Sfax	1– >60	24/30,573	0.08 (0.05–0.1)	*T. saginata*	Microscopy	[108]
Tunisia	01/1996–12/2012	Tunis	on	4/20,033	0.02^p^ (0.005–0.05)	*T. saginata*	Microscopy	[109]
United Arab Emirates	01/2008–12/2009	Emirate of Sharjah (5 different hospitals)	1–58	0/10,514	0^q^ (0-0.04)	*Taenia* spp.	Microscopy	[110]
United Arab Emirates	01/2013–12/2013	Sharjah city	16– >44	31/21,347	0.15 (0.1–0.2)	*Taenia* spp.	Microscopy	[111]
Yemen	2009	Hadhramout Governorate (rural and urban areas)	6–13	9/600	1.5 (0.7–2.8)	*T. saginata*	Microscopy	[112]
Yemen	on	Sahar District	7–14	1/534	0.2 (0.005–1.0)	*T. saginata*	Microscopy	[113]

^a^Refers to Thais working in Israel

^b^Study refers to Jewish, Bedouine and Ethiopian children living in southern Israel. *Taenia* spp. infections were only detected in Ethiopian children and were most probably *T. saginata* infections according to the authors

^c^Most probably a *T. saginata* case due to dietary restrictions

^d^Refers to the period 1997–1998

^e^Refers to the period 2007–2008

^f^Two different numbers reported regarding *Taenia* spp. prevalence in the publication

^g^Children from regions where raw wastewater is used for irrigation

^h^Children from regions where wastewater irrigation is not practiced

^i^Most probably *T. saginata* cases due to dietary restrictions

^j^Exact prevalence rate not mentioned. Estimated from graph (see figure 5 in reference [49])

^k^Refers to expatriate workers from Philippines, Indonesia, Indian sub-continent and Africa (food handlers and housemaids)

^l^Refers to expatriate workers from different countries. Highest infection rates with *T. saginata* observed among Indonesian, Indian, Bangladeshis, Filipinos and Pakistanis. Not mentioned how differentiation of *Taenia* species was made, although faeces were checked for presence of gravid proglottids

^m^Refers to expatriate workers from Asia and Africa

^n^Refers to Asian female house keepers from Indonesia, Sri Lanka, Philippines and Thailand

^o^Refers to Sudanese food handlers

^p^Refers to food handlers

^q^64% of the samples were from expatriates and the rest 36% were from native Emiratis. The expatriate population was a heterologous mixture of various nationalities from Indian subcontinent, Middle East, Southeast Asia, Africa and east European countries

*Abbreviations*: na, not available; CI, confidence interval; Ref, Reference

### Bovine cysticercosis

Prevalence data from Egypt (7 records) and Israel (1 record) were found upon database screening or elsewhere (Table 3). Data from Egypt derived from six different governorates situated along the Nile and a large-scale study included data from all official abattoirs (6,160,982 slaughtered cattle and buffaloes from 1994 to 1997). An additional large-scale study from an abattoir in the south of Israel provided prevalence data over a considerable study period (1973–2007) and number of slaughtered cattle, i.e. 629,549 animals. For the remaining 19 MENA countries, data on the prevalence of bovine cysticercosis could not be obtained, even from Sudan that has one of the highest cattle populations globally. However, as previously mentioned, the parasite has apparently been eradicated from the Republic of Cyprus [25].

**Table 3:** Prevalence of bovine or buffalo cysticercosis

Country	Time-frame	Location of study	No. of positive animals/No. of animals tested	Prevalence (95% CI)	Technical diagnosis	Reference
Egypt	01/1994–12/1997	All official abattoirs	4885/2,124,629	0.2^a^ (0.2-0.2)	Meat inspection	[26]
			36,201/499,610	7.3^b^ (7.1-7.3)		
			4902/3,536,743	0.1^c^ (0.1–0.1)		
Egypt	05/2006–06/2007	Assiut Governorate	8/510	1.6^d^ (0.7-3.0)	Meat inspection	[29]
			2/268	0.7^c^ (0.1-2.7)		
Egypt	09/2014–05/2015	El-Minia Governorate	20/100	20.0^d^ (12.7–29.1)	Meat inspection	[114]
Egypt	on	Cairo Governorate	3/75	4.0^d^ (0.8–11.2)	Meat inspection	[115]
			22/75	29.3^d^ (19.4–41.0)	Ab-ELISA	
Egypt	03/2010–02/2013	Gharbia Governorate	50/11,281	0.4^d^ (0.3–0.6)	Meat inspection	[116]
			24/19,089	0.1^c^ (0.08–0.2)		
Egypt	01/2014–12/2014	Qalyubia Governorate	313/3450	9.1^c^ (1.0–10.0)	Meat inspection and molecular confirmation	[24]
Egypt	08/2015–07/2016	Aswan Governorate	3433/45,780	7.5^d^ (7.3-7.7)	Meat inspection	[27]
			3/223	1.3^c^ (0.3-3.9)		
Israel	1973–2007	Marbek Abattoir, Qiryat Mal'akhi	2568/629,549	0.4 (0.4–0.4)	Meat inspection	[28]

^a^Native cattle

^b^Imported cattle

^c^Buffaloes

^d^Cattle

*Abbreviations*: na, not available; CI, confidence interval

Although meat inspection-based prevalence data are provided in the eight published studies on bovine cysticercosis, in two of the studies from Egypt, antibody-ELISA (infection prevalence of 29.3%) and molecular identification of tissue cysts by PCR, sequencing and phylogenetic analysis were also performed. Bovine cysticercosis prevalence was determined for both cattle and buffaloes in five studies from Egypt, only for cattle in one study from Egypt and one from Israel, and one study from Egypt solely focused on buffaloes. Bovine cysticercosis prevalence for cattle based on meat inspection ranged between 0.2–20%. For buffaloes, lower prevalence values, ranging between 0.1–9.1% were observed. Interestingly, three studies [26–28] reported considerably higher infection rates in imported than native cattle. In Israel this was connected to extensive import of cattle from Australia after 1998 (more than 500,000 imported cattle between 1998 and 2007, 95% of which originated from Australia), which seems to have contributed to cysticercosis outbreaks, whereas in a study from Egypt all imported animals were of Sudanese origin. In addition, two studies reported higher infection rates in older animals, particularly females [27, 29].

**Fig. 3:**
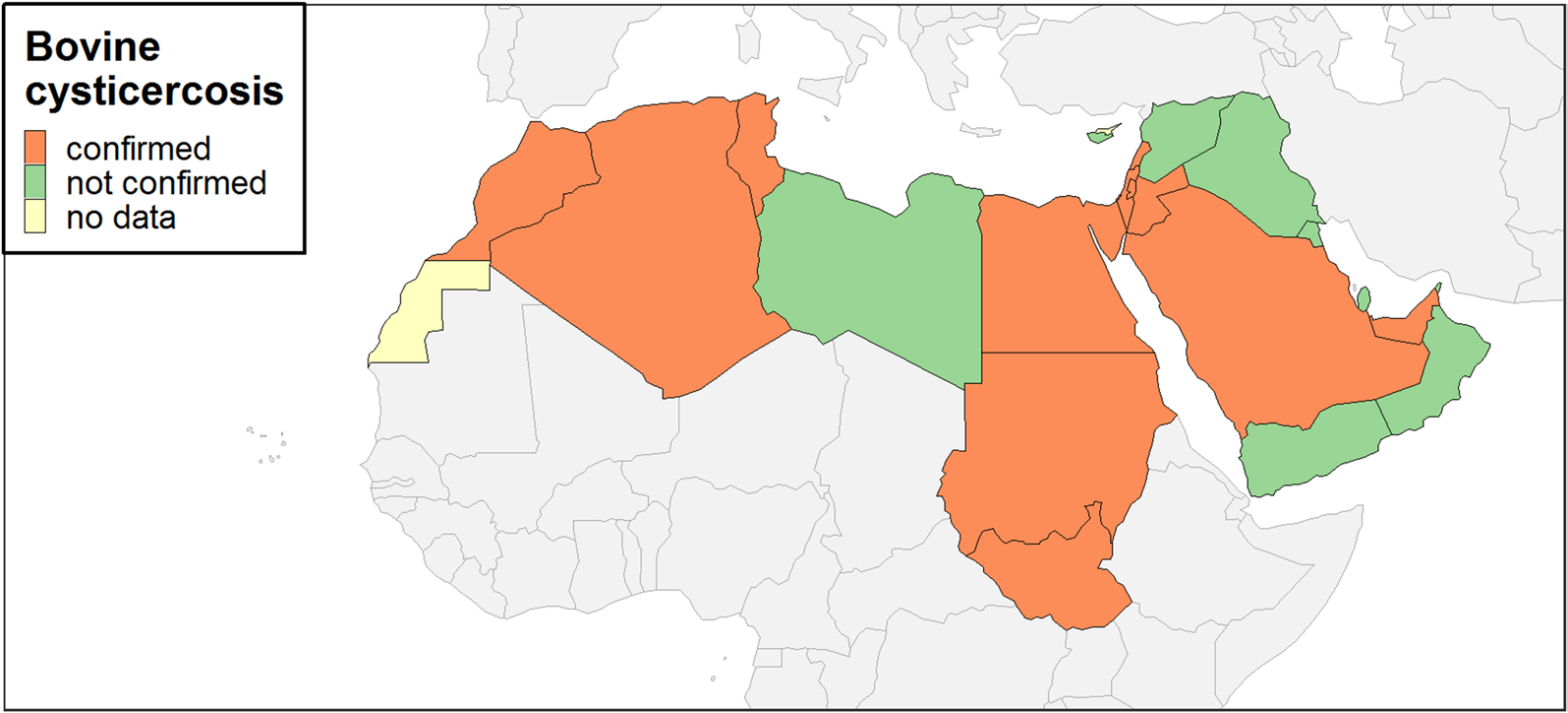
Bovine cysticercosis occurrence based on OIE reports and countries with studies reporting prevalence in the period 1990–2017

In addition to Egypt and Israel, bovine cysticercosis presence could be further confirmed based on OIE reports for the following countries: Algeria, Jordan, Lebanon, Morocco, Palestine, Saudi Arabia, Sudan/South Sudan (data after South Sudan became independent in 2011 were not available), Tunisia and UAE (Table 4). Therefore, bovine cysticercosis presence could be confirmed for 12 out of the 21 MENA countries (Fig. 3).

**Table 4:** Bovine cysticercosis occurrence and number of cases, if provided, based on OIE data

Country/territory	1996	1997	1998	1999	2000	2001	2002	2003	2004	2005
Algeria^a^	3	2	+	on	on	on	on	on	on	on
Cyprus^a^	on	on	on	on	on	on	on	on	on	on
Egypt^a^	216	235	15,072	98	172	2692	698	164	3642	270
Iraq	on	on	on	on	on	on	on	on	on	−
Israel^a^	+	26	+	on	on	on	on	20	+	−
Jordan^a^	+	+	+	−	−	−	−	−	−	−
Kuwait^a^	−	−	−	−	−	−	−	−	−	−
Lebanon	+	+	+	+	on	on	on	−	−	+
Libya	−	−	−	−	−	−	−	−	−	−
Morocco	+	+	+	+	+	+	+	+	+	+
Oman	on	on	on	on	on	on	on	−	−	on
Palestine^a^	on	on	on	on	on	on	on	5	1	−
Qatar	on	on	on	on	on	−	−	−	−	−
Saudi Arabia	−	−	−	on	on	on	+		−	−
Sudan/South Sudan	+	on	on	on	on	on	on	−	−	−^b^
Syria	on	on	on	−	−	−	−	−	−	−
Tunisia	+	on	on	on	on	on	on	on	on	−
United Arab Emirates	+	+	on	+		−	−	−	−	on
Western Sahara	on	on	on	on	on	on	on	on	on	on
Yemen	on	−	on	on	on	on	on	on	on	on

^a^Notifiable disease

^b^Refers to Sudan

*Key*: +, reported present or known to be present; −, disease absent (date of last outbreak not known); na, not available

For Iraq, Kuwait, Libya, Oman, Syria and Yemen, although unspecified *Taenia* spp. or *T. saginata* infections in humans were reported, OIE data do not indicate the presence of cysticercosis, although for some of those countries there is a considerable degree of underreporting. Neither *T. saginata*/*Taenia* spp. nor bovine cysticercosis records were found for Qatar and Western Sahara.

## Discussion

The sparsity of data on *T. saginata* taeniosis and bovine cysticercosis in the MENA region prompted us to summarize existing knowledge. Based on data gathered through this systematic review, *T. saginata* taeniosis is definitely present across the MENA region, being reported in just over 50% (11/21) of the countries studied. Furthermore, human infections with unspecified *Taenia* spp. were found for an additional seven countries; thus, taeniosis occurs in most (86%; 18/21) of the countries of the MENA region. Because both pig farming and pork consumption are uncommon in many of the MENA countries (over 90% of the local population were registered as Muslim or Jewish in 2010 [30]), it is likely that unspecified taeniosis cases are mainly due to *T. saginata*, as was noted in some of the publications. However, recent data from the Arabian Peninsula indicate the presence of autochthonous *Taenia solium* transmission [31], potentially resulting in human neurocysticercosis. In addition, porcine cysticercosis due to *T. solium* has been detected by meat inspection in 0.09% of slaughtered pigs in Egypt [26]. Some countries of the Arabian Peninsula are attractive destinations for millions of economic immigrants, some of whom come from *T. solium*- and/or *Taenia asiatica*-endemic countries, such as from south-/southeast Asia and sub-Saharan Africa [11, 32–35]; thus, *T. solium* or *T. asiatica* taeniosis cannot be entirely excluded. As the results presented herein relied almost exclusively on microscopy for monitoring parasitic infections in general, it is important to note that species differentiation by the application of appropriate methods, such as multiplex real-time PCR, would be essential in case of a history of pork consumption, given the morphological similarity between *Taenia* spp. eggs [36, 37].

Diagnosis of intestinal parasites typically relies on the microscopic detection of transmission stages in human faecal samples which was also the result of our search, despite the enormous inter- and intra-country disparities (e.g. rural *vs* urban areas) in terms of healthcare infrastructure. Such data often result, especially to what taeniosis infections concerns, in prevalence underestimation because of the poor sensitivity of microscopy (e.g. due to the intermittent excretion of eggs) and the asymptomatic nature of infection (many infected individuals never get tested) [38]. The observed prevalence range (0.02–8.6%) based on microscopy studies conducted in the MENA region is comparable to that reported for southern/eastern Africa and the Americas, but the prevalence values were higher than those for the Russian Federation, western and eastern Europe [39–43]. However, comparison between studies is challenging due to their variability in design (variable factors including, for example, duration of study, inclusion of only certain target/age groups, different diagnostic methods used, randomization of participants, geographical coverage). The adoption of a consensus protocol for taeniosis surveillance purposes by clinical investigators such that bias is minimized is therefore highly recommended, as previously suggested [40].

The present review clearly demonstrates the lack of bovine cysticercosis-related epidemiological data and data on its possible economic impact for the MENA region. Meat inspection-based prevalence studies were available only for Egypt and Israel, confirming considerable infection rates, especially in imported cattle, but also native cattle and buffaloes. Moreover, bovine cysticercosis could be confirmed for a further 10 countries in the MENA region based on OIE data, thus demonstrating the presence of this infection in just over 50% of the countries considered, despite the widespread distribution of taeniosis in the region. Apart from a possible underestimation in the reported prevalence values for both Egypt and Israel due to the low sensitivity of visual meat inspection [44, 45], lack of data and underreporting to OIE for the remaining countries strongly reflect the actual inconsistency in reporting systems. This might be attributed to the fact that bovine cysticercosis is not notifiable in many of those countries and to OIE.

The MENA region covers a wide diversity of environments, from wet coastal regions to high mountain plateaus and arid steppes and deserts in the interior. Around 2% of the region is considered to consist of humid areas and over 6% of the population live in these areas, while most of the region (85%) is considered to be arid or semi-arid and approximately 23% of the population live in these areas [46]. The remaining population lives in both urban centers and intensively irrigated agricultural areas of the arid and semi-arid parts of the MENA region, where bovine rearing might also take place. Large-scale irrigated systems are primarily situated in Morocco and Libya, and along the Nile (South Sudan, Sudan, Egypt), Euphrates and Tigris rivers (Syria, Iraq). In addition, water scarcity in the region (only 1% of global freshwater resources are available in the MENA region) may be addressed by extensive wastewater reuse [46]. Such wastewater may not always be treated sufficiently so that all pathogens are efficiently eliminated; on average 43% of wastewater is treated in the MENA region [47]. For instance, a study from Morocco demonstrated *Taenia* spp. infections in children from areas where untreated wastewater irrigation was practiced, but this was not the case in control areas [48]. In some territories, such as in Palestine, sewage channels are often open, and thus prone to flooding [49, 50]. This may increase the risk of animals coming into contact with pathogens in human sewage, such as *Taenia* eggs, and cattle or buffaloes contracting bovine cysticercosis [49]. Even in cases (e.g. in Tunisia) where sewage/wastewater is treated, *Taenia* spp. eggs could not be efficiently eliminated [51], whereas in some cases *Taenia* spp. eggs were even found in drinking water, such as in Iraq [52]. Considering both the significant cattle and buffalo population, as well as the specific geographic, environmental and demographic characteristics of the area, efforts should be directed towards obtaining more detailed prevalence-based data by considering relevant aspects on the human, animal and eco-system interface from a One-Health perspective. This would constitute the basis for the development of models predicting possible spatiotemporal transmission clusters and high-risk areas.

Globalization poses an increased threat for the spread of, among others, foodborne pathogens, including the agents of cysticercosis/taeniosis *via* the international movement of people, animals, and their products, and potentially contaminated produce or other fomites from endemic regions. This was also evident for the MENA region, where the import of live cattle from Australia to Israel after 1998 seems to have contributed to cysticercosis outbreaks in this country [28]. Additionally, the prevalence of cysticercosis infection was higher in imported cattle than native cattle in two studies from Egypt [26, 27]. Sudan and Brazil were the biggest suppliers of live cattle to Egypt during 2017 (approximately 250,000 head, mainly intended for immediate slaughter), whereas exports of chilled beef from the USA to Lebanon reached a value of $3.2 million in 2015 [53, 54]. Given that the cysticercosis infection rates in Brazilian cattle range from very low levels to 18.8% [40], import of such high numbers implies that some infected cattle will be imported. The role of mass religious gatherings, such as the annual Hajj pilgrimage to Mecca in Saudi Arabia, where thousands of live animals, including cattle, are imported from various neighboring countries, slaughtered, and prepared for consumption, poses both a real zoonotic risk and a considerable challenge for local veterinary and medical authorities [6, 55]. The role of such socio-cultural events in the epidemiology of taeniosis should not be underestimated and deserves further attention. It was, for instance, previously demonstrated that the Eid al-Adha (the second of two great Muslim festivals, the other being Eid al-Fitr) celebration in Kosovo might contribute to an increased annual incidence of canine echinococcosis [56]. Certain culinary habits from the MENA region, potentially promoting *T. saginata* infection, include the consumption of raw, smoked, salted or dried beef products, with the most characteristic representative of the Lebanese and Levantine (Levant is an approximate historical geographical term, referring to a large area in the Eastern Mediterranean) cuisine being “Kibbeh nayyeh”, which is prepared using minced raw beef [57, 58]. A further example of how cysticercosis epidemiology could potentially be affected by globalization is also the recent boycott against Qatar by neighboring countries. This has stimulated a massive import of cattle from various other countries in order for the country to cover its milk needs [59]. The above facts highlight the need for the development of sensitive diagnostic tests that efficiently detect infected animals or carcasses, and evaluation of their application in the international live cattle and chilled beef trades [20, 44, 60]. Currently, apart from meat inspection, only antigen-based ELISA tests are capable of detecting infective (live) cysticerci, and the reduced sensitivity in light infections may mean that some infections would go undetected [20, 44, 45]. Harmonization of the legislation scheme underlying international bovine/beef trade with regard to ensuring only the entrance of bovine cysticercosis-free chilled meat in the food chain/or live animals, would be an additional necessary act complementary to the respective national preventive, antemortem, and post mortem control measures.

## Conclusions

The present review demonstrates the widespread distribution of *T. saginata* taeniosis in the MENA region. However, both prevalence and distribution data, as well as economic burden data, on bovine cysticercosis are largely unavailable. Therefore, complementary to the application of appropriate control measures covering the whole spectrum of “primary production-to-consumption” food chain continuum, efforts should be directed towards obtaining more detailed epidemiological data for both *T. saginata* taeniosis and bovine cysticercosis. This would enable identification of probable transmission routes by considering possible risk factors (such as wastewater reuse and animal trade). *Taenia saginata* control and elimination offers ground for an integrated “One Health” approach, thus interdisciplinary collaboration between health, agricultural, and environmental authorities of all countries in the MENA countries should be further encouraged. Epidemiological evidence to support decisions on appropriate interventions to be applied could be significantly improved by such an approach.

## Additional files

**Additional file 1.** PRISMA checklist.

**Additional file 2.** Search protocol.

## Abbreviations

B.C.: Before Christ
CI: Confidence interval
IMEMR: Index Medicus for the Eastern Mediterranean Region
MENA: Middle East and North Africa
GOOSE: World Organization for Animal Health/Office International des Epizooties
UAE: United Arab Emirates
WHO: World Health Organization
