# Supplementary material for: Epidemiology of Taenia saginata taeniosis/cysticercosis: a systematic review of the distribution in the Middle East and North Africa
Source: Parasit Vectors. 2019 Mar 15;12:113. doi: 10.1186/s13071-019-3339-5 (PMC6419812; doi:10.1186/s13071-019-3339-5)
Supplement: Supplementary file 2 — Additional file 2. Search protocol. [file 13071_2019_3339_MOESM2_ESM.docx]

**Additional file 2**

**Epidemiology of Taenia saginata taeniosis/cysticercosis: a systematic review of the distribution in the Middle East and North Africa**

**Search protocol**

**Aim:** To synthesize the current evidence on presence/absence and prevalence (where reported) of *T. saginata* (taeniosis/cysticercosis) in the Middle East and North Africa region (MENA)

**Questions to answer:**

Which countries have reported the presence of *T. saginata* in cattle and/or humans since 1990?

For which countries are prevalence data available and what is the quality of that data?

Are specific geographical locations available for these data?

**Methods:**

The review will be conducted in line with the PRISMA statement 2009 (<http://www.bmj.com/content/339/bmj.b2700#alternate>) and will include each item on the PRIMSA checklist <http://www.prisma-statement.org>.

Articles will be selected for inclusion into the systematic review through the identification of all potentially relevant citations through the search strategy. The citations within identified articles will also be included in the screening process. Duplicates will be excluded, followed by screening of titles and abstracts with articles excluded if they do not explicitly report occurrence or prevalence of *T. saginata*. Full text articles will then be screened for exclusion criteria with those remaining utilised in the review.

Full text articles will then be read and relevant data extracted and entered into a Microsoft Excel spreadsheet.

**Databases:**

- PubMed (<https://www.ncbi.nlm.nih.gov/pubmed/>)
- OpenGrey (<http://www.opengrey.eu>)
- WHO IRIS (<http://apps.who.int/iris/>)
- Index Medicus for the Eastern Mediterranean Region (IMEMR) (<http://www.emro.who.int/information-resources/imemr-database/>)
- HANDISTATUS II (<http://web.oie.int/hs2/report.asp?lang=en>)
- WAHIS

([http://www.oie.int/wahis_2/ public/wahid.php/Diseaseinformation/statusdetail](http://www.oie.int/wahis_2/%20public/wahid.php/Diseaseinformation/statusdetail))

**Search term (Pubmed):** (cysticerc* OR cisticerc* OR C. bovis OR taenia* OR tenia* OR saginata OR taeniosis OR teniosis OR taeniasis OR ténia OR taeniid OR cysticerque ) AND (Algeria OR Egypt OR Libya OR Morocco OR Sudan OR South Sudan OR Tunisia OR Western Sahara OR Yemen OR United Arab Emirates OR Syria OR Saudi Arabia OR Qatar OR Palestine OR Israel OR Oman OR Lebanon OR Kuwait OR Jordan OR Iraq OR Cyprus)

**Inclusion/exclusion:**

- **Exclusion criteria:**
  - studies concerning a different parasite than *T. saginata*
  - studies reporting data outside from study area
  - studies reporting/using data older than 1990 or published after December 31^st^ 2017
  - studies reporting results out of the scope of the review questions
  - duplicated data
- **Languages:** All
- **Year data collection:** 1^st^ January 1990 – 31^st^ December 2017
- **Geographical range:** All countries within the Middle East and North Africa region
